# A Sufficient Condition for Graphic Sequences with Given Largest and Smallest Entries, Length, and Sum *

**DOI:** 10.23638/DMTCS-20-1-25

**Published:** 2018

**Authors:** Brian Cloteaux

**Affiliations:** National Institute of Standards and Technology, Applied and Computational Mathematics Division, USA

**Keywords:** degree sequence

## Abstract

We give a sufficient condition for a nonnegative integer list to be graphic based on its largest and smallest elements, length, and sum. This bound generalizes a result of Zverovich and Zverovich.

We denote a finite list of nonnegative integers, *α* = (*α*_1_,...,*α*_*n*_) where *α*_1_ ≥ ... ≥ *α*_*n*_ as a *degree sequence*. A degree sequence is said to be *graphic* if it corresponds to the set of edge adjacency values of the nodes for some simple graph. In 1960, Erdős and Gallai gave a complete characterization of the set of˝ graphic degree sequences.

## **Theorem 1** (Erdős and Gallai (1960)).

*Let α* = (*α*_1_,...,*α*_*n*_) *be a nonincreasing degree sequence. Then the degree sequence α is graphic if and only if the sum of α is even and for each integer k where* 1 ≤ *k* ≤ *n,*

(1) $$\sum_{i=1}^{k}\alpha_{i}\leq k(k-1)+\sum_{i=k+1}^{n}\min\left\{k,\alpha_{i}\right\}.$$

It was later shown by I. Zverovich and E. Zverovich that some degree sequences with bounded largest and smallest elements can be verified to be graphic based on their length.

## **Theorem 2** (Zverovich and Zverovich (1992), Theorem 6).

*Let α* = (*α*_1_,...,*α*_*n*_) *be a nonincreasing degree sequence of positive integers with even sum. If*

(2) $$n\geq \frac{{\left(\alpha_{1}+\alpha_{n}+1\right)}^{2}}{4\alpha_{n}},$$

*then α is graphic.*

There have been several extensions to the original result of Zverovich and Zverovich. These results have been either specializations, such as when the gaps between consecutive integers in the sequence are bounded (Barrus et al. (2012)), or by sharpening the bound for specific sequences (Cairns et al. (2015); Cairns and Mendan (2016)). In this article, we show the following generalization of the Zverovich and Zverovich result by taking into account the sequence sum.

## Theorem 3.

*Let α* = (*α*_1_,...,*α*_*n*_) *be an integer sequence such that n* − 1 ≥ *α*_1_ ≥ ... ≥ *α*_*n*_ ≥ 0 *and with even sum* $s=\sum_{i=1}^{n}\alpha_{i}$ *where nα*_1_ *> s > nα*_*n*_. *If*

(3) $$\left(\alpha_{1}-\alpha_{n}\right)\left(\frac{n-\alpha_{1}-1}{n\alpha_{1}-s}+\frac{\alpha_{n}}{s-n\alpha_{n}}\right)\geq 1,$$

*then α is graphic.*

## 1 Proof of Theorem 3

Before providing a proof of this result, we state some needed definitions and theorems. The *complement* of a degree sequence *α* = (*α*_1_,*α*_2_,...,*α*_*n*_) is the sequence $\bar{\alpha }=\left(n-\alpha_{n}-1,\ldots ,n-\alpha_{1}-1\right)$. It is straightforward to see that *α* is graphic if and only if $\bar{\alpha }$ is graphic. It is important to note that Equation (3) is invariant under complementation, meaning that

(4) $$\left(\alpha_{1}-\alpha_{n}\right)\left(\frac{n-\alpha_{1}-1}{n\alpha_{1}-s}+\frac{\alpha_{n}}{s-n\alpha_{n}}\right)=\left({\bar{\alpha }}_{1}-{\bar{\alpha }}_{n}\right)\left(\frac{n-{\bar{\alpha }}_{1}-1}{n{\bar{\alpha }}_{1}-\bar{s}}+\frac{{\bar{\alpha }}_{n}}{\bar{s}-n{\bar{\alpha }}_{n}}\right),$$

where $\bar{s}=\sum_{i=1}^{n}{\bar{\alpha }}_{i}$.

A second definition is that a sequence *α majorizes* a second sequence *β* = (*β*_1_,...,*β*_*n*_), where both sequence have the same length and sum, if and only if

(5) $$\sum_{i=1}^{k}\alpha_{i}\geq \sum_{i=1}^{k}\beta_{i},$$

for each *k* from 1 to *n*.

Majorization is a partial order over the set of degree sequences with identical sum. Our use of majorization stems from the following result.

## **Theorem 4** (Ruch and Gutman (1979), Theorem 1).

If the degree sequence α is graphic and α majorizes β, then β is graphic.

In addition, we use the following two results that reduce the number of Erdős-Gallai inequalities needed˝ to verify that a sequence is graphic. These are found in Tripathi and Vijay (2003).

## **Theorem 5** (Tripathi and Vijay (2003), Theorem).

*For the degree sequence α* = (*α*_1_,...,*α*_*n*_), *define the set of indices* I *such that i* ∈ I *if α*_*i*_ *> α*_i+1_. *For Theorem 1, it suffices to check only the inequalities for the indices in* I.

## **Theorem 6** (Tripathi and Vijay (2003), Lemma).

*For the degree sequence α* = (*α*_1_,...,*α*_*n*_), *define the set of indices* I *where i* ∈ I *if α*_*i*_ ≥ *i* − 1. *For Theorem 1, it suffices to check only the inequalities for the indices in* I.

We are now ready to prove Theorem 3.

## Proof of Main Theorem:

We begin by defining the set D as the set of all degree sequences that satisfy the hypotheses conditions for *α*_1_, *α*_*n*_, *n*, and *s*. We then define the sequence *α′* ∈ D as

(6) $$\alpha^{\prime }=\left(\underset{p}{\underset{︸}{\alpha_{1},\ldots ,\alpha_{1}}},\gamma ,\underset{n-p-1}{\underset{︸}{\alpha_{n},\ldots ,\alpha_{n}}}\right),$$

where *α*_1_ *> γ* ≥ *α*_*n*_ and *s* = *pα*_1_ + *γ* + (*n* − *p* − 1)*α*_*n*_. From the condition that *nα*_1_ *> s > nα*_*n*_, it follows that the sequence *α′* cannot be regular, i.e., *α*_1_ *> α*_*n*_. To show that the sequence *α′* exists, we construct it by first defining the value of *γ* as *γ* = *τ* + *α*_*n*_ where *τ* is the solution to the congruence equation

(7) $$\tau \equiv s-n\alpha_{n}\;\left(\mathrm{mod}\;\left(\alpha_{1}-\alpha_{n}\right)\right),$$

that satisfies 0 ≤ *τ < α*_1_− *α*_*n*_. Since *α*_1_ *> α*_*n*_, then *τ* is well-defined. From definition of *τ*, the value of *γ* is *α*_*n*_ ≤ *γ < α*_1_. It follows from the congruence equation that *s* − *nα*_*n*_ − *τ* = *p*(*α*_1_ − *α*_*n*_) for some integer *p*. To show that the sequence $\alpha^{\prime }$ can be constructed, we simply need to show that 0 *< p < n*. First, we rewrite the value for *p* as

(8) $$p=\frac{s-n\alpha_{n}-\tau }{\alpha_{1}-\alpha_{n}}.$$

From 0 ≤ *τ < α*_1_− *α*_*n*_, *α*_*n*_ ≤ ... ≤ *α*_1_, and $\sum_{i=1}^{n}\alpha_{i}=s<n\alpha_{1}$, then

(9) $$0<\frac{\alpha_{1}-\alpha_{n}-\tau }{\alpha_{1}-\alpha_{n}}\leq \frac{\alpha_{1}+(n-1)\alpha_{n}-n\alpha_{n}-\tau }{\alpha_{1}-\alpha_{n}}\leq \frac{s-n\alpha_{n}-\tau }{\alpha_{1}-\alpha_{n}}<\frac{n\alpha_{1}-n\alpha_{n}-\tau }{\alpha_{1}-\alpha_{n}}\leq n,$$

implying 0 *< p < n* and establishing that the sequence $\alpha^{\prime }$ does uniquely exists.

We observe that the sequence *α′* majorizes all the other sequences in D. If there would exist a sequence *β* ∈ D where *α′* does not majorize *β* then there would exist an index *k* where $\sum_{i=1}^{k}\beta_{i}>\sum_{i=1}^{k}\alpha_{i}^{\prime }$. The first index where this could occur is at *k* = *p* + 1, since the first *p* values of *α*^0^ are the maximum possible value α_1_. If $\sum_{i=1}^{p+1}\beta_{i}>\sum_{i=1}^{p+1}\alpha_{i}^{\prime }$, then $\sum_{i=p+2}^{n}\beta_{i}<\sum_{i=p+2}^{n}\alpha^{\prime }=(n+p-1)\alpha_{n},$ forcing a value in the sequence *β* to be less than *α*_*n*_ and violating its membership in the set D. There is α_*n*_ similar argument for remainder of the indices, establishing that *α′* must majorize all the members of D. It follows from Theorem 4 that if α′ is graphic then every sequence in D must also be graphic. Thus we need only to consider whether the sequence *α′* is graphic in order to prove the result.

We also make the assumption concerning *α′* that $\alpha_{1}\geq n-\alpha_{n}-1={\bar{\alpha }}_{1}.$ This assumption holds in general because if $\alpha_{1}<{\bar{\alpha }}_{1}$ then we simply use the complement of *α′* for our argument since Equation (3) is invariant under complementation.

Now let us examine the Erdős-Gallai (EG) inequalities forα′. We apply Theorem 5, to show that if the EG inequalities hold at *k* = *p* and *k* = *p* + 1 in order to prove that the sequence α′ is graphic. First consider the case when *p < α*_*n*_. The resulting EG inequality for *k* = *p* is

(10) $$p\alpha_{1}\leq p(p-1)+p(n-p)=p(n-1),$$

which is false only when *α*_1_ *> n*−1, but we assumed that *α*_1_ ≤ *n*−1 ensuring that this inequality always holds. There is an identical argument for the case when *k* = *p* + 1 establishing that the EG inequalities always hold when *p < α*_*n*_.

Now consider when the case when *p* ≥ *α*_*n*_. By first multiplying both sides of the Inequality (3) by (*s*−*nα*_*n*_)(*nα*_1_−*s*)*/*(*α*_1_−*α*_*n*_)^2^, we algebraically manipulate this new inequality to derive the following equivalent expression,

(11) $$\frac{1}{4}{\left(1+\alpha_{1}+\alpha_{n}\right)}^{2}-n\alpha_{n}\leq \frac{{\left(2s-n(n-1)+\left(n-\alpha_{n}-1\right)\left(n-\alpha_{n}\right)-\alpha_{1}\left(\alpha_{1}+1\right)\right)}^{2}}{4{\left(\alpha_{1}-\alpha_{n}\right)}^{2}}.$$

Combining the facts that *α*_1_ ≥ *n* − *α*_*n*_ − 1 along with *s* ≥ *pα*_1_ + (*n* − *p*)*α*_*n*_, this new Inequality (11) becomes

(12) $$\frac{1}{4}{\left(1+\alpha_{1}+\alpha_{n}\right)}^{2}-n\alpha_{n}\leq \frac{{\left(2s-n(n-1)+\left(n-\alpha_{n}-1\right)\left(n-\alpha_{n}\right)-\alpha_{1}\left(\alpha_{1}+1\right)\right)}^{2}}{4{\left(\alpha_{1}-\alpha_{n}\right)}^{2}}\;\leq \frac{{\left(2\left(p\alpha_{1}+(n-p)\alpha_{n}\right)-n(n-1)+\left(n-\alpha_{n}-1\right)\left(n-\alpha_{n}\right)-\alpha_{1}\left(\alpha_{1}+1\right)\right)}^{2}}{4{\left(\alpha_{1}-\alpha_{n}\right)}^{2}}\;={\left(p-\frac{1}{2}\left(1+\alpha_{1}+\alpha_{n}\right)\right)}^{2}.$$

We now rewrite the Inequality (12) as

(13) $$p\alpha_{1}\leq p(p-1)+(n-p)\alpha_{n},$$

implying *pα*_1_ ≤ *p*(*p* − 1) + *γ* + (*n* − *p* − 1)*α*_*n*_, which is precisely the EG inequality for *α′* at *k* = *p*.

For *k* = *p* + 1, we split the instance into two cases: *p* ≥ *γ* and *p < γ*. If *p < γ*, then we use Theorem 6, which states that we only need to check to the largest index *k* such that *α*_*k*_ ≥ *k* − 1, in order to prove that a sequence is graphic. Thus, if *p < γ*, then satisfying the EG inequality for *k* = *p* already established that the sequence is graphic. Else, if *p* ≥ *γ*, then *γ* ≤ 2*p* − *α*_*n*−1_ and summing this inequality with the Inequality (13), we derive *pα*_1_+*γ* ≤ (*p*+1)*p*+(*n*−(*p*+1))*α*_*n*_, which is the EG inequality for *k* = *p*+1. Therefore, the premise is established.

## 2 Remarks About The Bound

This result generalizes the bound of Zverovich and Zverovich. We observe that for the right hand side of Inequality (11),

(14) $$\frac{{\left(2s-n(n-1)+\left(n-\alpha_{n}-1\right)\left(n-\alpha_{n}\right)-\alpha_{1}\left(\alpha_{1}+1\right)\right)}^{2}}{4{\left(\alpha_{1}-\alpha_{n}\right)}^{2}}\geq 0.$$

It follows that if $\frac{1}{4}{\left(\alpha_{1}+\alpha_{n}+1\right)}^{2}-n\alpha_{n}\leq 0$ then the sequence *α* is graphic by Theorem 3. By rearranging this inequality, we derive the bound of Theorem 2.

In general, this bound is sharp in the sense that there exist sequences that satisfy the inequality, but any modification to parameters that causes the inequality to be not satisfied allows for non-graphic sequences to be formed. For example, consider the following set of degree sequences: for a given value of *α*_1_, define *α*_*n*_ = 2, *n* = *α*_1_ + 1, and *s* = 4*α*_1_ − 2. These values satisfy the Inequality (3) with *A* equality, and so any sequence with these parameters is graphic. We notice that modifying any one of these values so that the inequality is no longer satisfied while keeping the other three fixed allows for non-graphic sequences. For example, if we increase *α*_1_ by 1 or decrease *n* by 1, we have sequences, such as $\text{as}\;\left(\alpha_{1}+1,\alpha_{1}-1,\underset{\alpha_{1}-1}{\underset{︸}{2,\ldots ,2}}\right)$ and $\left(\alpha_{1},\alpha_{1},4,\underset{\alpha_{1}-3}{\underset{︸}{2,\ldots ,2}}\right)$ where the largest value in the sequence is larger than number of remaining values. Likewise, if we decrease *α*_*n*_ by 1 or increase the sum by 2 (in order to preserve an even sum), then we have sequences such as $\left(\alpha_{1},\alpha_{1},3,\underset{\alpha_{1}-3}{\underset{︸}{2,\ldots ,2}},1\right)$ and $\left(\alpha_{1},\alpha_{1},4,\underset{\alpha_{1}-2}{\underset{︸}{2,\ldots ,2}}\right)$ that again can be quickly verified to be non-graphic.

In comparison to the Zverovich and Zverovich result, there is a slight difference in the requirements for applying the bound. This bound requires that *α*_1_ ≤ *n* − 1 and this restriction cannot be relaxed. This is because there are sequences where *α*_1_ *> n* − 1 but fulfill the inequality (such as (8,6,6,6,6,6,6,6)). In contrast to Zverovich and Zverovich result, if that inequality is satisfied, it implies that *α*_1_ ≤ *n*−1 (proof of Theorem 1.2, Cairns and Mendan (2016)).

It should be noted that this bound cannot be used for sets of regular sequences, i.e., *α*_1_ = *α*_*n*_, but we can handle this case by using in conjunction the result that states if *α*_1_ − *α*_*n*_ ≤ 1, then *α* is graphic (Chen (1988), Lemma 1). In addition, we note that when we extract the smallest value in the sequence, we should choose the smallest value where *α*_*i*_ *>* 0. This is because zeros in a degree sequence do not change whether the entire sequence is graphic or not and can be discarded.

Finally, this result has practical use when coupled with the usual Erdős-Gallai conditions for checking˝ if a sequence is graphic. Before a sequence can be tested using the EG conditions, it must first be sorted. During this sorting step, the values for largest and smallest indices, sequence length, and sequence sum can be easily extracted. Thus, this new condition may save a number of steps in graphic testing for certain sequences by verifying that a sequence is graphic without having to compute the full EG inequalities (Cloteaux (2015)).

## References

[R1] Barrus MD, Hartke SG, Jao KF, and West DB. Length thresholds for graphic lists given fixed largest and smallest entries and bounded gaps. Discrete Math., 312(9):1494–1501, 2012 ISSN 0012365X. doi: 10.1016/j.disc.2011.05.001.

[R2] Cairns G and Mendan S. An improvement of a result of Zverovich-Zverovich. Ars Math. Contemp, 10(1):79–84, 2016 ISSN 1855–3966. URL http://amc-journal.eu/index.php/amc/article/view/451.

[R3] Cairns G, Mendan S, and Nikolayevsky Y. A sharp refinement of a result of Zverovich-Zverovich. Discrete Math, 338(7):1085–1089, 2015 ISSN 0012–365X. doi: 10.1016/j.disc.2015.02.001.

[R4] Chen YC. A short proof of Kundu’s *k*-factor theorem. Discrete Math., 71(2):177–179, 1988. doi: 10.1016/0012-365X(88)90070-2.

[R5] Cloteaux B. Is this for real? Fast graphicality testing. Computing in Science & Engineering, 17(6): 91–95, 2015. doi: 10.1109/MCSE.2015.125.

[R6] Erdős P and Gallai T. Graphs with prescribed degrees of vertices.˝ Mat. Lapok, 11:293–303, 1960.

[R7] Ruch E and Gutman I. The branching extent of graphs. J. Combin. Inform. System Sci, 4(4):285–295, 1979 ISSN 0250–9628.

[R8] Tripathi A and Vijay S. A note on a theorem of Erdős & Gallai. Discrete Math., 265(1–3):417–420, 2003 ISSN 0012–365X. doi: 10.1016/S0012-365X(02)00886-5.

[R9] Zverovich IÈ and Zverovich VÈ. Contributions to the theory of graphic sequences. Discrete Math., 105(1–3):293–303, 1992 ISSN 0012–365X. doi: 10.1016/0012-365X(92)90152-6.

